# Surgical Resection of Retroperitoneal Aggressive Angiomyxoma: Case Report and Review of the Literature

**DOI:** 10.7759/cureus.1485

**Published:** 2017-07-18

**Authors:** Mircea Beuran, Cezar Ciubotaru, Alexandru Runcanu, Valentin Enache, Ionut Negoi

**Affiliations:** 1 Department of General Surgery, Emergency Hospital of Bucharest, Romania; 2 CH Iii, Emergency Hospital of Bucharest, Romania; 3 Pathology Department, Emergency Hospital of Bucharest, Romania

**Keywords:** aggressive angiomyxoma, soft tissue tumor, surgical resection

## Abstract

Aggressive angiomyxoma is a benign stromal tumor with a higher prevalence in middle-aged women. The objective of this case report is to illustrate the aggressive clinical behavior of this benign tumor. We present the case of a 45-year-old female patient, with tumor recurrence after multiple surgical resections of a pelvis-subperitoneal angiomyxoma. Surgical excision of the tumor, with extensive pelvic dissection and organ resection, was performed. The 12-month follow-up showed no tumor recurrence. Based on this case, and the published literature we may conclude that surgical resection represents the main treatment of aggressive angiomyxoma, even though it is associated with significant morbidity and a poor local control of the tumor.

## Introduction and background

Angiomyxoma is a soft tissue neoplasia [1], with the highest prevalence in young white middle-aged women [2]. It is a rare tumor, with a slow growth rate, and despite property to invade adjacent structures, the metastasis rate is low [3]. Although described for the first time by Virchow in 1883 as soft tissue myxomata, fewer than 350 cases of aggressive angiomyxoma were published in the medical literature [4]. The most important clinical feature of this type of tumor is the high local recurrence rate [5]. On pathology examination, the vascular structures are well represented, and differential diagnosis with other myxoid tumors is difficult [6]. The surgical approach should be the first step of treatment in this type of disease [7]. The aim of this case report is to reveal the aggressive clinical behavior of this benign tumor. We obtained the patient’s informed consent for publication.

## Review

Case report

A 45-year-old woman, with multiple surgical resections (2007, 2009, 2010, and 2014 – tumor resection and left oophorectomy) of a pelvis-subperitoneal angiomyxoma, was referred to our hospital one year after the latest surgical procedure for a tumor recurrence.

On clinical examination, a large tumor could be palpated into the hypogastrium, with a bulging in the right gluteal and ischioanal areas in standing position. A 3/3 cm elastic tumor bulged into the right femoral area. The pelvic magnetic resonance imaging (MRI) revealed a well-circumscribed tumor located into the right pararectal, parametrial, paravaginal, and iliac areas, with extension medial to the femoral vein into the femoral area; inhomogeneous with the presence of solid and fluid density areas; without lymph node involvement.

**Figure 1:**
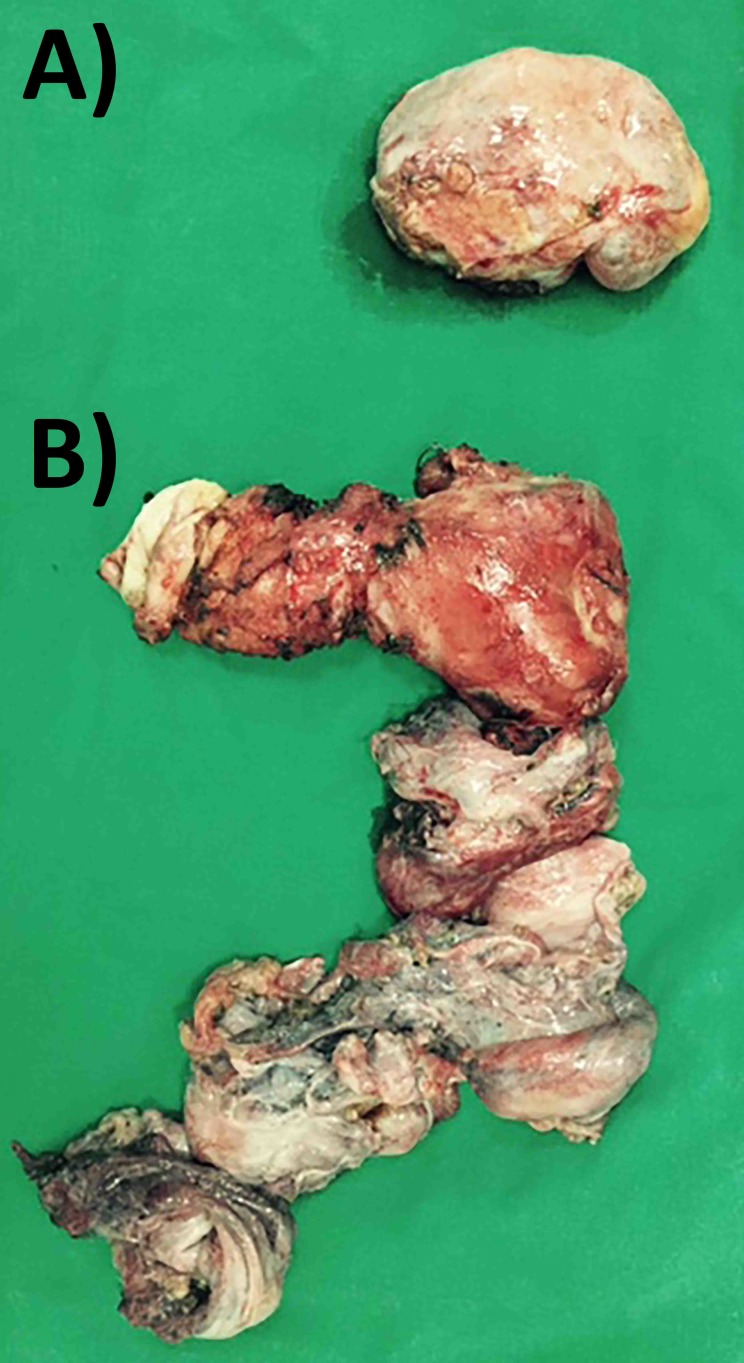
Resection specimen, including the left ovary (A) and recurrent tumor, uterus and both adnexa, and a 3-cm segment of the right ureter (B).

Total hysterectomy with bilateral oophorectomy, wedge rectal resection, and four centimeters of the right pelvic ureter resection was performed (Figure 1).

Postoperative evolution was favorable, without postoperative complications, with discharge on postoperative day 11. Two months later, synchronous with right ureter stent removal, tumor extension into the right femoral area was resected, with no complications.

The 12-month follow-up showed no tumor recurrence

**Figure 2:**
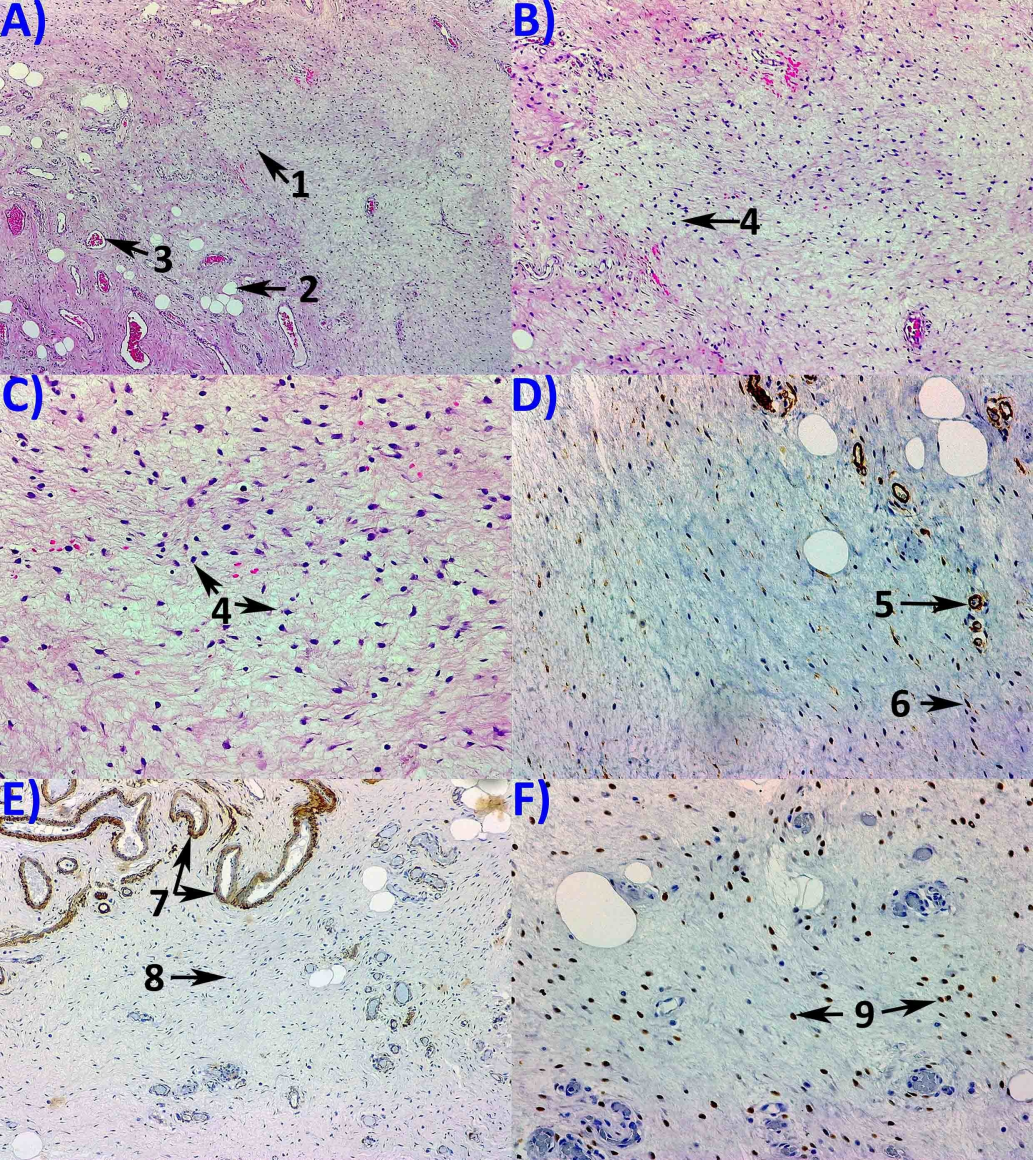
Pathology examination. (A) Hematoxylin and Eosin staining using 50x objective revealing tumor proliferation with scarce tumor cells (1); adipocytes (2) and blood vessels (3) can be observed; no tumor necrosis is seen. Hematoxylin and Eosin staining using 100x (B) and 200x (C) objectives, showing fusiform and stellate cells (4) with low mitotic activity, with abundant stroma. Immunohistochemistry expression using 200x objective revealing of CD 34 positivity in the tumor cells (5) and blood vessels (6) (D); 100x objective revealing muscle-specific actin (MSA) positivity in the muscular layer of blood vessels (7) but negative in the tumor cells (8) (E); 200x objective revealing ER positivity in the tumor cells (F).

Histopathological examination revealed aggressive angiomyxoma with estrogen receptors positivity in 75% of the tumor cells, progesterone receptors in 80% of the tumor cells, actin and CD34 positivity in blood vessels, desmin positivity, and Ki67 positivity in 3% of the cells (Figure 2).

**Figure 3:**
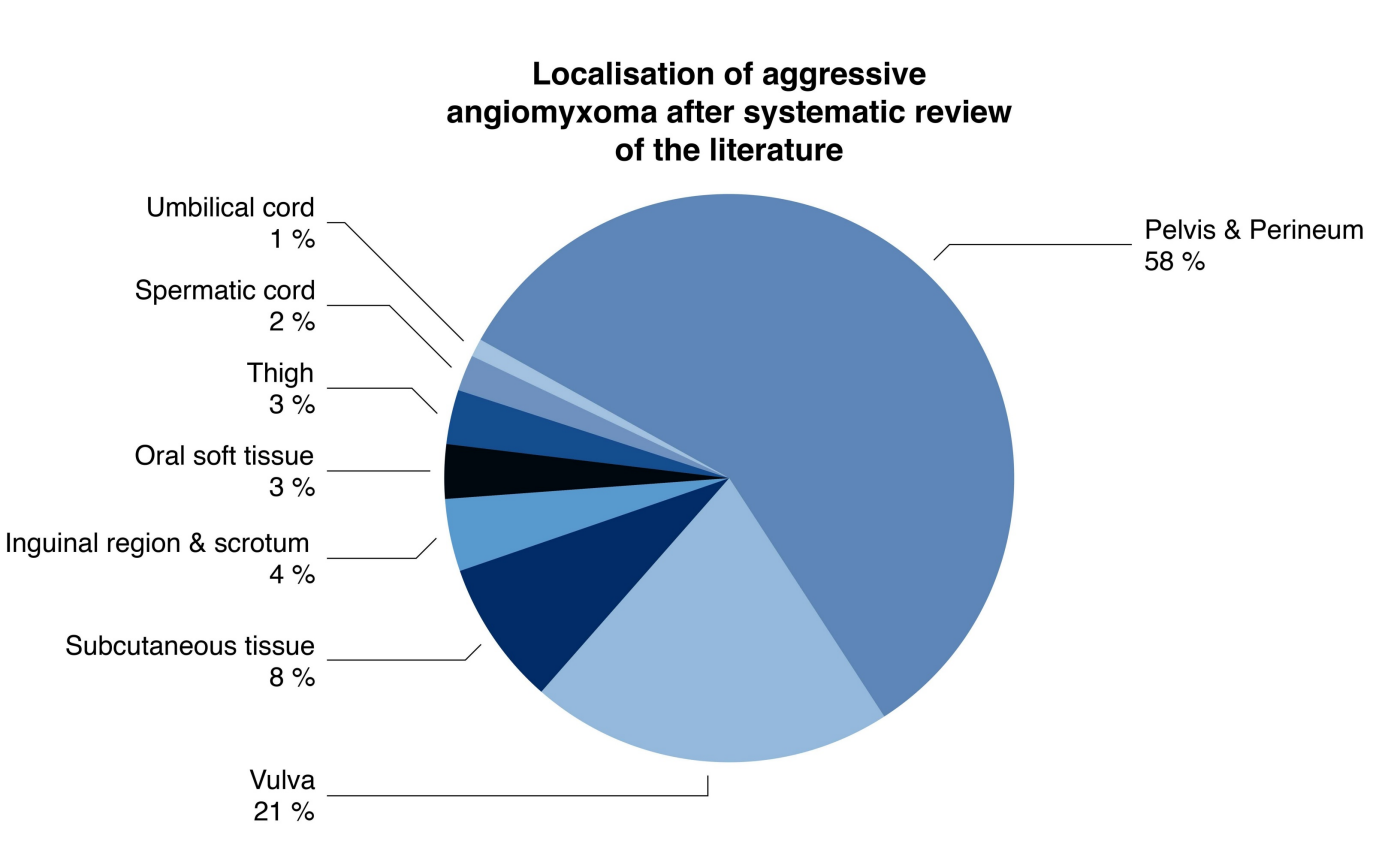
Localization of aggressive angiomyxoma according to the peer-reviewed literature.

Review of the literature (Figure 3).

Discussions

Steeper and Rosai coined this tumar as aggressive angiomyxoma, the distinctive variant of myxoid neoplasm that occurred predominantly in female pelvic or perineal areas [8]. Although described in both gender, the prevalence of aggressive angiomyxoma is 6.6 higher in women compared to men [5]. Aggressive angiomyxoma is considered to be a low-grade sarcoma, with the property to recur locally [9]. Two cases of metastatic aggressive angiomyxoma were described [4].

The histological structure of aggressive angiomyxoma consists mainly in a bland myxoid and vascular stroma, without a capsule, being locally infiltrative. The tumor macroscopic aspect is similar to that of healthy tissue; therefore the complete surgical resection is difficult [10]. A primitive pluripotent cell located around the vessels of connective tissue was proposed as the origin of the aggressive angiomyxoma [11]. Overlapping immunohistochemical and ultrastructural features between vulvar angiomyxoma, aggressive angiomyxoma, and angiomyofibroblastoma were revealed [11]. Our patient had immunohistochemical similarities with ones showed by Alameda, et al., with CD34 positivity, and muscle-specific actin (MSA) negativity [11].

As the aggressive angiomyxoma usually develops during the fertile period and pregnancy, they are believed to have hormone receptors [12]. The current evidence shows the constant positivity of the tumor cells for estrogen and progesterone receptors [4]. In our case, the immunohistochemistry examination revealed estrogen and progesterone receptors positivity in 75% and 80% of the tumor cells, respectively.

The diagnosis of this type of tumor is usually difficult, due to clinical onset of unspecific symptoms; it consists in local pain, swelling, dyspareunia, bowel and urinary tract dysfunctions. The aggressive angiomyxomas present a characteristic pattern on MRI, with ‘swirls’ aspect of low-intensity internal stranding on T1- and T2-weighted images [13-15].

The main treatment of aggressive angiomyxoma is surgery, with the aim to obtain R0-type resection. Recently, minimally invasive laparoscopic and robotic-assisted were described [16-17]. However, this is difficult to be obtained, the texture and appearance of the tumor being similar to that of healthy tissues [18]. Chan, et al. presented five cases, two of them with significant operative morbidity: one with division and reimplantation of the right ureter, blood loss of 3500 ml, and the second with bladder injury and 5000 ml blood loss [5]. They reviewed 106 cases of aggressive angiomyxoma and found a 47% local recurrence rate, 71% of recurrences occurring during the first three years [5]. Han-Geurts, et al. reviewed their experience of seven patients with aggressive angiomyxoma, three being pregnant at the time of diagnosis [12]. They questioned the benefits of radical surgery, which did not seem to significantly reduce the recurrence rate when compared to R1 resections [12]. Sozutek, et al. presented a case of giant aggressive angiomyxoma of the pelvis misdiagnosed as incarcerated femoral hernia [19]. Our case had a similar extension of the pelvic tumor through the femoral ring into the femoral area. Sanfilippo, et al. presented a multicenter retrospective analysis of 31 patients with aggressive angiomyxoma [20]. Twenty-nine patients had at least one surgical resection (nine R2-type). The two-year local recurrence rate in patients with R0/R1 resection was 21%. Thirty-five percent of patients received hormonal therapy, which was able to obtain the complete response in a subset of patients and disease control in all cases [20].

Hormonal therapy, including tamoxifen, raloxifene, and gonadotropin-releasing hormone (GnRH) may be useful to downsize the tumor preoperatively or as adjuvant therapy [21]. On the other hand, prolonged GnRH treatment in these young patients is associated with significant osteoporosis or depression [22].

The effectiveness of radiotherapy and chemotherapy as adjuvant therapy to control local recurrences is unclear, due to the low mitotic activity [23].

## Conclusions

Aggressive angiomyxoma is a benign stromal tumor with the capacity to invade adjacent structures and high rate of local recurrence. Surgery represents the mainstay of treatment; however, it should be done with minimal morbidity. Adjuvant therapies, such as hormonal suppression therapies, to control local recurrence should be considered.
